# Tumour-stroma ratio and 5-year mortality in gastric adenocarcinoma: a systematic review and meta-analysis

**DOI:** 10.1038/s41598-019-52606-7

**Published:** 2019-11-05

**Authors:** Niko Kemi, Maarit Eskuri, Joonas H. Kauppila

**Affiliations:** 1 Cancer and Translational Medicine Research Unit, Medical Research Center, University of Oulu and Oulu University Hospital, Oulu, Finland; 2 Upper Gastrointestinal Surgery, Department of Molecular medicine and Surgery, Karolinska Institutet, Karolinska University Hospital, Stockholm, Sweden

**Keywords:** Cancer microenvironment, Surgical oncology

## Abstract

Tumour-stroma ratio (TSR) is a novel potential prognostic factor in cancers and based on the proportions of stroma and tumour area. The prognostic value of TSR in gastric cancer is incompletely known. The aim of this study was to estimate prognostic significance of TSR in gastric adenocarcinoma. A search of PubMed (MEDLINE), Web of Science, EMBASE, Cochrane and Scopus databases was performed. A meta-analysis was conducted on five-year survival in gastric cancer patients using inverse variance random-effects methods. The literature search yielded 5329 potential titles, of which a total of seven studies were eligible. Results of six studies including a total of 1779 patients were pooled in the meta-analysis. Only 23 (1.3%) of the patients received neoadjuvant therapy. All six studies had a cut-off of 50% for the proportion of stroma when dividing the patients into low- and high stroma groups. Low TSR (high amount of stroma) was strongly associated with increased five-year mortality (hazard ratio 2.19, 95% CI 1.69–2.85). In conclusion, TSR is a strong prognostic factor in gastric cancer. It could be used to estimate prognosis of gastric cancer patients not receiving neoadjuvant chemotherapy. Further studies including patients receiving neoadjuvant therapy are recommended.

## Introduction

Gastric cancer is the third most common cause of cancer death in the world^1^. The survival after the surgery of gastric cardia cancer has been improved during the past decades, while in non-cardia gastric cancer the improvement in survival been more modest^2^. TNM-classification for cancers provides prognostic information based on the degree of tumour progression, but does not take into account the tumour biology, and we still see recurrences and cancer death after surgery even in early-stage gastric cancer^3^. Additional, easy-to-replicate histological factors that could identify gastric cancer patients with highest risk of recurrence or mortality are needed.

Some tumour biology-related factors, such as tumour-stroma ratio (TSR), have been proposed to identify patients with high risk of cancer mortality. TSR is defined as the area of stroma compared to area of the tumour cells in the tumour and is strongly associated with survival in several cancer types, including colorectal cancer^4^, breast cancer^5^, and hepatocellular carcinoma^6^. TSR can be easily and routinely analysed from haematoxylin-eosin (HE) stained slides routinely used for diagnostic purposes^7^. Tumours that have high amount of stroma have low TSR, and tumours that have low amount of stroma have high TSR.

Some studies have suggested that low TSR is associated with poor survival in gastric cancer^8–10^. Despite that the prognostic impact of TSR in gastric cancer is currently poorly known. The aim of this meta-analysis was to identify all studies on tumour-stroma ratio in and estimate the prognostic value of TSR in gastric adenocarcinoma.

## Methods

This study was a meta-analysis conducted according to the PRISMA guidelines^11^. The study followed a study protocol established a priori.

### Search

The literature search was conducted in August 2018 using a keyword search on PubMed (MEDLINE), Web of Science, EMBASE, and Cochrane databases using the terms (stroma* OR Glasgow tumor microenvironment score) AND (gastri* OR gastrectomy OR gastroesophageal OR gastro-oesophageal OR oesophagogastric OR esophagogastric) AND (neoplas* OR cancer OR carcinoma OR adenocarcinoma) AND (prognos* OR mortality OR survival). Scopus database was searched using terms (stroma*) AND (gastri* OR gastrectomy OR gastroesophageal OR gastro-oesophageal OR oesophagogastric OR esophagogastric) AND (neoplas* OR cancer OR carcinoma OR adenocarcinoma) AND (prognos* OR mortality OR survival).

### Study selection

The studies considered for inclusion had to be original articles written in English. They had to contain assessment of proportion of intratumoural stroma compared to tumour area and contain hazard ratios for survival, or Kaplan-meier curves stratified by intratumoural stromal proportion.

Duplicates of studies identified in literature search were removed. Titles of studies left after removing duplicates were screened by one researcher, and studies that clearly did not fill the inclusion criteria were excluded. Abstracts of the studies left after reading titles were read by one researcher, and the studies clearly not fulfilling the inclusion criteria were excluded. If the study fulfilled the criteria or there was not enough information in the abstract to exclude the study, full texts of the articles were studied independently by two researchers. If there were disagreements, the studies were discussed with third researcher and consensus was reached.

### Data extraction

The data necessary for this study were extracted independently by two researchers from the original studies. The data collected included the name of the first author, the study interval, the type of the study, the number of patients in the study, the country of the study population, the age and sex of patients included in the study, if the patient received chemotherapy or not and characteristics of the tumour the patient had (histological type, histological grade and TNM-stage). Study quality was assessed independently by the two researchers using the Newcastle-Ottawa scale, as included studies were cohort studies. Discrepancies on study quality were settled by consulting third researcher.

### Definition of exposure and outcome

The exposure of this study was high amount of intratumoural stroma. The patient group with low amount of intratumoural stroma (high TSR) was considered as control group. The primary endpoint of this study was death during the five-year follow-up period after surgery. The primary outcome of this study was 5-year overall survival, as it is commonly used as outcome in studies considering prognosis in gastric cancer. The secondary outcome of this study was overall survival. The endpoint for overall survival was death during follow-up after surgery.

### Statistical analysis

Review Manager 5.3 (The Cochrane Collaboration, Oxford, UK) was used to perform statistical analyses. The estimates of hazard ratios for 5-year mortality and overall mortality were extracted from the studies, with preference to estimates from multivariable analyses to reduce confounding. If multivariable analysis was not available, univariable analysis was used instead. If the data was presented only as a Kaplan-Meier curve, the WebPlotDigitizer tool (http://arohatgi.info/WebPlotDigitizer) was used to extract proportion of surviving patients on 12, 24, 36, 48 and 60 months for both patient groups when estimating five-year mortality. When estimating overall mortality, the longest follow-up period available was used and datapoints were extracted every 12 months until the end of follow-up. Natural logarithms of hazard ratios and standard errors were then calculated based on those measurement points, according to the method described by Tierney *et al*.^12^ Generic inverse variance-method with random-effect models was used to calculate estimates of average hazard ratios with Review Manager. Publication bias was estimated by inspecting the funnel plots instead of statistical testing, given the small number of included studies^13^.

### Ethics approval

No ethical approval was sought for this systematic review and meta-analysis.

## Results

### Study selection

**Figure 1:**
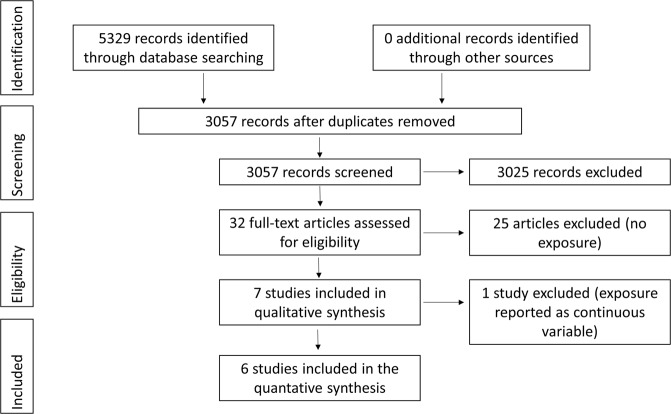
PRISMA diagram depicting the selection process of studies included in the meta-analysis.

The study selection is summarized in Fig. 1. Some 5329 titles were found in the literature search, and 3057 of them were left after removing duplicates. After the exclusion of studies not fulfilling the inclusion criteria based on title and abstracts there were 32 titles left, of which full text versions were studied and seven of them filled the inclusion criteria. Of these, a Singaporean study compared the lowest tertile to the highest tertile of tumour stroma measured with computerized method without reporting any percentage cut-offs, and could not be included in the meta-analysis due to different methodology compared to the other studies^14^. The details of the six studies fulfilling the inclusion criteria are presented in Table 1.

**Table 1:** Characteristics and quality of the studies included in the qualitative synthesis.

Study	Country	Study interval	Assessment	Type of study	Number of patients	Study quality
Ahn *et al*.^15^	South-Korea	2009–2012	Retrospective	Cohort	196	poor (6/9)
Aurello *et al*.^8^	Italy	2004–2015	Retrospective	Cohort	106	good (7/9)
Kemi *et al*.^9^	Finland	1983–2016	Retrospective	Cohort	583	good (8/9)
Lee *et al*.^17^	South-Korea	2005–2008	Retrospective	Cohort	175	fair (7/9)
Peng *et al*.^10^	China	2002–2011	Retrospective	Cohort	494	good (7/9)
Wu *et al*.^14^	Singapore	n.d.	Retrospective	Cohort	131 + 153*	fair (6/9)
Zhou *et al*.^16^	China	2000–2011	Retrospective	Cohort	225	good (7/9)

n.d., not described; *two independent cohorts were studied.

### Included studies

The six studies selected included a total of 1779 patients. In all studies TSR was assessed from the resected specimen. Five of the included studies assessed the proportion of stroma from HE-stained slides^8–10,15,16^, while one of the studies based its analysis of proportion of stroma on immunohistochemical stainings^17^. All of the studies used 50% of stroma as cut-off between low and high TSR groups. One of the studies only included patients with diffuse type carcinoma^17^. One study included only patients with T-grade 3 or 4 Tumours^15^. Two of the studies included some patients operated with palliative intent^9,15^. Only one of the studies included patients with neoadjuvant chemotherapy^9^. Further details of patients included in the studies are presented in Table 2 and 3.

**Table 2:** Patient characteristics in studies included in the qualitative synthesis stratified by tumour-stroma ratio.

Study	Number of patients		Age of patients		Sex ratio (M:F)		Neoadjuvant therapy	
	High TSR	Low TSR	High TSR	Low TSR	High TSR	Low TSR	High TSR	Low TSR
Ahn *et al*.^15^	118	78	52 ≤ 60 years 66 > 60 years	42 ≤ 60 years 36 > 60 years	85:33	42:36	None	None
Aurello *et al*.^8^	41	65	mean 70,1 years		57:49		None	None
Kemi *et al*.^9^	241	342	Mean 69.3 years	Mean 65.0 years	154:87	198:144	5/241	17/342
Lee *et al*.^17^	111	64	56 ≤ 50 years 55 > 50 years	40 ≤ 50 years 24 > 50 years	66:45	28:36	None	None
Peng *et al*.^10^	254	240	Median 58,8 years	Median 59,1 years	183:71	166:74	None	None
Wu *et al*.^14^ cohort LS-2	131		Total median 68 years		Total 81:50		None	
Wu *et al*.^14^ cohort SG-3	153		Total median 65 years		Total 95:53, 5 unknown		Total 1/153	
Zhou *et al*.^16^	139	86	Total 99 < 60 years, 126 ≥ 60 years		Total 168:57		None	None

TSR, tumour-stroma ratio; n.d., not described.

**Table 3:** Tumour characteristics in studies included in the qualitative synthesis stratified by tumour-stroma ratio.

Study	Histological type		Histological grade of differentiation		Tumour stage	
	High TSR	Low TSR	High TSR	Low TSR	High TSR	Low TSR
Ahn *et al*.^15^	Intestinal 68 Diffuse 38 Mixed 12	Intestinal 23 Diffuse 47 Mixed 8	Well- or moderate 64 Poor 54	Well- or moderate 20 Poor 58	T3: 75* T4: 43*	T3: 40* T4: 38*
Aurello *et al*.^8^	n.d.	n.d.	n.d.	n.d.	I-II: 15 III: 26	I-II: 35 III: 30
Kemi *et al*.^9^	Intestinal 174 Diffuse 62 Mixed 5	Intestinal 119 Diffuse 208 Mixed 15	n.d.	n.d.	I-II: 190 III-IV: 51	I-II: 169 III-IV: 173
Lee *et al*.^17^	Diffuse (111)	Diffuse (64)	n.d.	n.d.	T1-2: 53* T3-4: 58*	T1-2: 10* T3-4: 54*
Peng *et al*.^10^	AC 206 Mucinous or SRC 38 Others 7	AC 205 Mucinous or SRC 28 Others 6	Well- or moderate 60 Poor 146	Well- or moderate 70 Poor 135	I-II: 103 III: 151	I-II: 75 III: 165
Wu *et al*.^14^ cohort LS-2	Total Intestinal 91 Diffuse 30 Mixed 10		Total Well- or moderate 62 Poor 68 Unknown 1		Total I-II: 89 III: 41 Unknown: 1	
Wu *et al*.^14^ cohort SG-3	Total Intestinal 72 Diffuse 59 Mixed/unclassifiable 22		Total Well- or moderate 55 Poor 91 Undifferentiated/unknown 7		Total I-II: 52 III-IV: 98 Unknown: 3	
Zhou *et al*.^16^	Total Intestinal 100 Diffuse 125		Total Well or moderate 100 Poor 125		Total I-II: 108 III: 117	

^*^T-stage due to no TNM-stage provided;

TSR, tumour-stroma ratio; n.d., not described; AC, adenocarcinoma; SRC, signet ring-cell carcinoma.

### Study quality

One of the studies received six points, four seven points and one eight points from quality assessment using Newcastle-Ottawa scale (Table 1). One of the studies provided multivariate hazard ratio with 95% confidence interval for five-year survival^9^. One of the studies provided univariate hazard ratio with 95% confidence interval for five-year survival^16^. Four of the studies didn’t provide hazard ratios for five-year survival but they provided Kaplan-Meier figures for overall survival^8,10,15,17^, which were used to calculate hazard ratios and 95% confidence intervals for five-year survival.

### Tumour-stroma ratio and 5-year mortality

**Figure 2:**
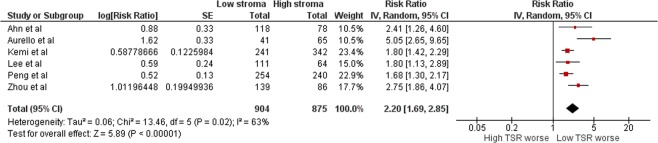
Forest plot comparing five-year survival in low and high TSR groups.

The meta-analysis was conducted on five-year survival data. The forest plot is shown in Fig. 2. The hazard ratio for stroma-rich group compared to stroma-poor group was 2.19 (95% CI 1.69–2.85). The statistical heterogeneity was high (I^2^ = 63%). The inspection of funnel plot showed some asymmetry, mainly caused by the smallest study in the meta-analysis^8^, suggesting small-study effects and a risk of publication bias (Supplementary Fig. 1). However, the removal of this study from the meta-analysis in a sensitivity analysis had only small effect on the estimate, resulting in a HR of 1.93 (95% CI 1.62–2.29), and low statistical heterogeneity (I^2^ = 20%). The Singaporean study not included in the meta-analysis also suggested worse survival in patients in the stroma-rich groups^14^.

### Tumour-stroma ratio and overall mortality

The forest plot of the meta-analysis conducted on overall survival data is shown in Supplementary Fig. 2. The hazard ratio for stroma-rich group compared to stroma-poor group was 2.33 (95% CI 1.81–3.01). Similarly to meta-analysis of 5-year mortality, statistical heterogeneity was high (I^2^ = 57%), which was mainly caused by the smallest study included^8^. Removal of this study resulted in a HR of 2.13 (95% CI 1.72–2.63) with lower heterogeneity (I^2^ = 41%).

## Discussion

Based on this first systematic review and a meta-analysis on TSR in gastric cancer patients, those patients with low TSR experience on average worse outcomes than those with high TSR.

Some strengths and limitations should be considered before conclusions on the results. The strengths of this study include broad literature search and stringent inclusion criteria, resulting in inclusion of all potentially eligible quality studies. The studies included have been performed in Caucasian and Asian populations, which increases the applicability of the pooled results. Only one study included patients treated with neoadjuvant therapy and even it included only 23 patients that received neoadjuvant therapy^9^, which is known to cause fibrosis in tumours^18^. Therefore, the results are not applicable to patients undergoing neoadjuvant treatment.

The quality of the studies was mostly good, but loss to follow-up was not described in four of the studies^10,15–17^. As all of the studies are single-center studies, potential selection bias might reduce the applicability of the results. However, all the studies included had the same direction of the results, including the two cohorts in the Singaporean study that was excluded from the meta-analysis^14^, with similar effect sizes, suggesting low selection bias. Lastly, analysis of 5-year mortality adjusted for confounders was not available in five of the six studies, which implies some residual confounding in the pooled estimate. However, the pooled estimate was very similar to the only study adjusted for confounders^9^. For overall mortality, the adjusted hazard ratios were available for three studies^9,10,17^. Subgroup analyses or meta-regression could have provided additional ways of taking confounders into account, but neither could be performed. Meta-regression is recommended only if ten ore more studies are available, while the six included studies in the present meta-analysis were clearly too few for a reliable meta-regression^19^. Only one study provided subgroup analysis of patients with different histological types^9^ and only one study provided subgroup analysis of patients with different T-grades^17^, preventing any subgroup analyses. Small study effects were seen in funnel plot with the smallest study included^8^, but the sensitivity analysis without this study showed only small impact on the hazard ratio for five-year survival.

Other ways of measuring survival, progression-free survival (PFS) and disease-free survival (DFS), could not be analysed. None of the studies reported PFS. DFS was reported in three studies, but only one of them defined DFS as time before recurrence of the disease or death^17^, while the other two studies defined DFS as time before recurrence of the disease, excluding death^8,16^. Therefore, DFS could not be reliably analysed. However, due to the aggressive nature of gastric cancer, five-year- and overall survival should relatively accurately depict outcomes after surgery, while the assessment of PFS and DFS might not provide additional value to this study.

In recent years the knowledge on the stromal component of the tumours has increased^20–22^. Cancer associated fibroblasts (CAFs) present in tumour stroma seem to be capable of contributing in turning normal tumour growth suppressing stromal microenvironment to one supporting tumour growth^21^. CAFs contribute to tumour growth in several ways. For example they secrete cytokines that promote desmoplastic environment^21^, they might promote angiogenesis^23^ and they might contribute to the chemoresistance of the tumour^24^. Stroma-rich tumours might benefit more from the growth-promoting capabilities of tumour, which could explain the worse prognosis of patients with low TSR.

As the role of stroma in the growth of tumour has become more apparent, the interest in development of therapies that target stroma instead of tumour has grown^25,26^. For example, studies on antibodies against fibroblast growth factor, a signalling molecule for CAFs^27^, antibodies against transforming growth factor beta, a tumor growth enhancing factor produced by CAFs^28^, and T-cell immunotherapies targeting the stroma^29,30^ are currently being developed in preclinical settings. While therapies targeting the stroma have not yet reached clinical use, they might become important in the future. If such therapies become available, it would be interesting to assess whether patients with low TSR would benefit more from these therapies than those with high TSR.

Based on the results of this meta-analysis, TSR is an important prognostic factor in gastric cancer. As it can be easily analysed from HE-stained slides, it could be used to estimate prognosis of patients after surgery and it is one factor that could be considered when planning adjuvant therapy. As the prognostic significance of TSR for patients treated with neoadjuvant chemotherapy is currently unknown, TSR should not be used to estimate prognosis of patients that received neoadjuvant chemotherapy.

In the future, large and prospective studies would be useful to estimate the additional value of TSR in clinical use. Studying TSR in patients before and after undergoing neoadjuvant therapy in relation to prognosis is necessary to apply the results in the neoadjuvant-treated population. New treatments targeting stromal component of the tumour are under development^25,26^, and could further increase the value of assessing TSR in cancer patients.

In conclusion, this first meta-analysis on TSR and gastric cancer shows that TSR is a strong prognostic factor in gastric cancer. TSR could be easily used as an adjunct for biological aggressiveness when deciding on adjuvant treatment in postoperative gastric cancer patients that have not undergone neoadjuvant therapy.

## Supplementary information

Supplementary figures

## Data Availability

We are willing to share study data upon request.
