# Author Correction: Tumour-stroma ratio and 5-year mortality in gastric adenocarcinoma: a systematic review and meta-analysis

**DOI:** 10.1038/s41598-020-62004-z

**Published:** 2020-03-17

**Authors:** Niko Kemi, Maarit Eskuri, Joonas H. Kauppila

**Affiliations:** 1 Cancer and Translational Medicine Research Unit, Medical Research Center, University of Oulu and Oulu University Hospital, Oulu, Finland; 2 Upper Gastrointestinal Surgery, Department of Molecular medicine and Surgery, Karolinska Institutet, Karolinska University Hospital, Stockholm, Sweden

Correction to: *Scientific Reports* 10.1038/s41598-019-52606-7, published online 05 November 2019

This Article contains an error in Figure 2 where the hazard ratio of 2.19 is incorrectly given as a risk ratio of 2.20.

The correct Figure 2 appears below as Figure 1.

**Figure 1:**
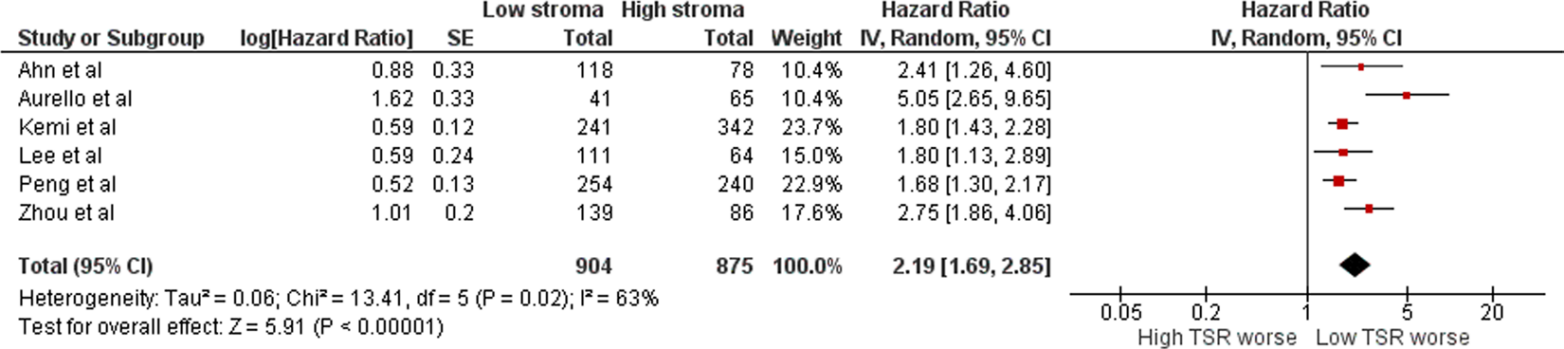
.

