# Supplementary material for: Tumour-stroma ratio and 5-year mortality in gastric adenocarcinoma: a systematic review and meta-analysis
Source: Sci Rep. 2019 Nov 5;9:16018. doi: 10.1038/s41598-019-52606-7 (PMC6831590; doi:10.1038/s41598-019-52606-7)

Tumor-stroma ratio and 5-year mortality in gastric adenocarcinoma: a systematic review and meta-analysis

Niko Kemi, Maarit Eskuri, Joonas H Kauppila

Supplementary figures

Supplementary figure 1. Funnel plot for assessment of publication bias

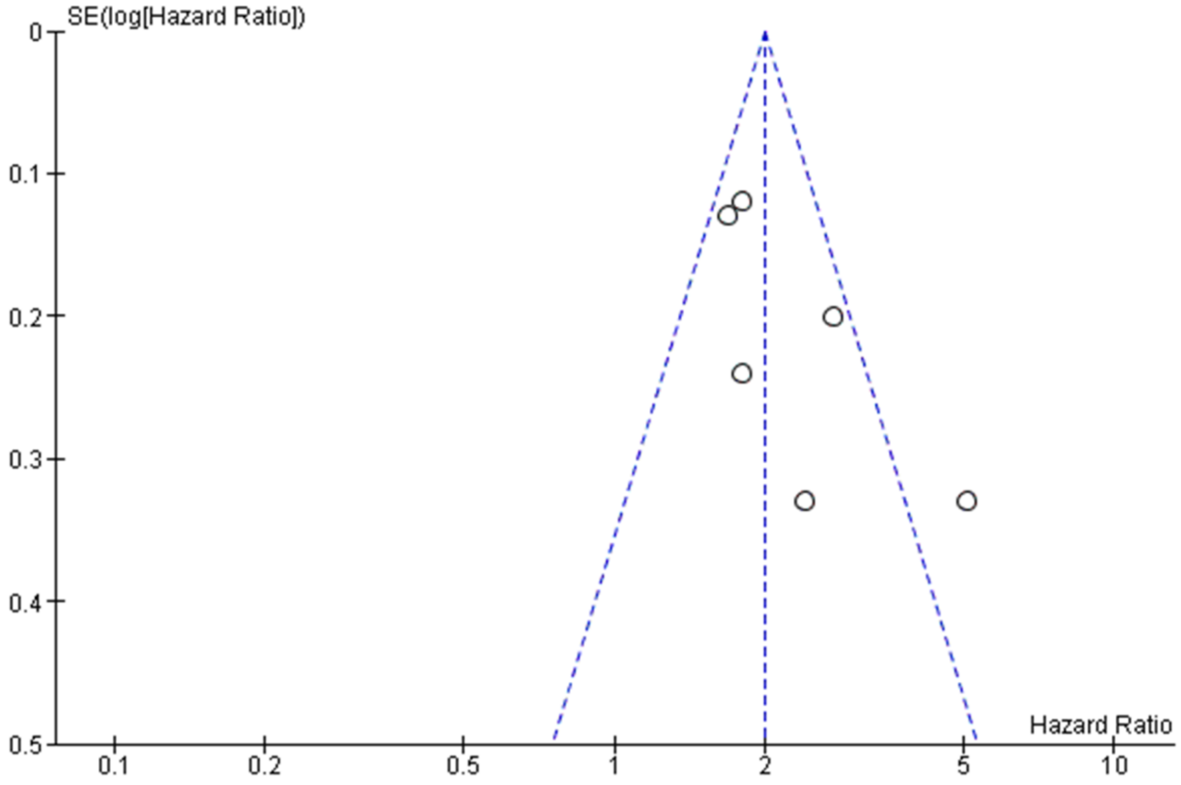

Supplementary figure 2. Forest plot comparing five-year survival in low and high TSR groups

Study or Subgroup	log[Hazard Ratio]	SE	Low stroma Total	High stroma Total	Weight	Hazard Ratio IV, Random, 95% CI
Ahn et al	1.12	0.33	118	78	10.6%	3.06 [1.61, 5.85]
Aurello et al	1.59	0.4	41	65	8.0%	4.90 [2.24, 10.74]
Kemi et al	0.54	0.11	241	342	26.5%	1.72 [1.38, 2.13]
Lee et al	0.92	0.27	111	64	13.6%	2.51 [1.48, 4.26]
Peng et al	0.65	0.15	254	240	22.8%	1.92 [1.43, 2.57]
Zhou et al	1	0.2	139	86	18.5%	2.72 [1.84, 4.02]
**Total (95% CI)**			**904**	**875**	**100.0%**	**2.33 [1.81, 3.01]**

Heterogeneity: Tau² = 0.05; Chi² = 11.60, df = 5 (P = 0.04); I² = 57%
Test for overall effect: Z = 6.51 (P < 0.00001)

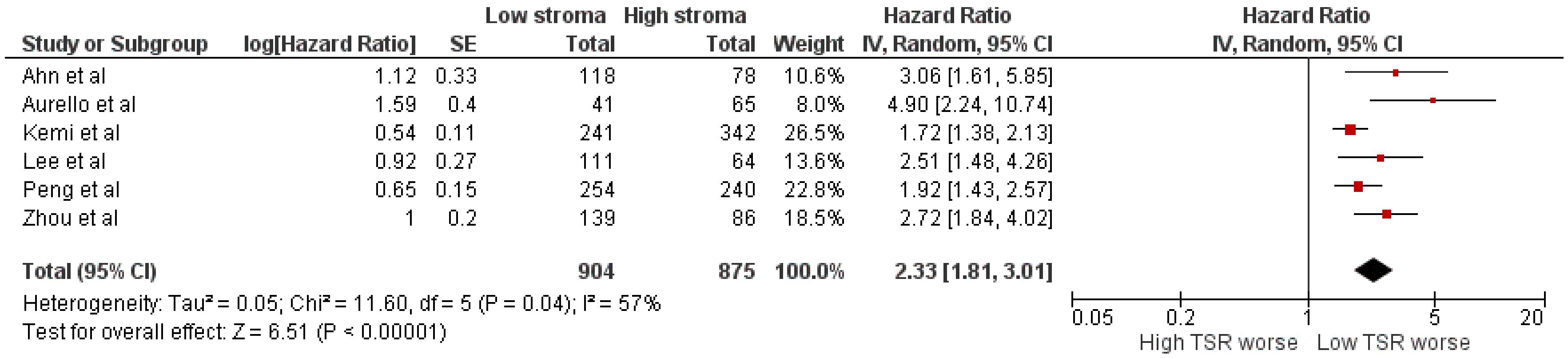

Supplement: Supplementary file 1 — Supplementary figures [file 41598_2019_52606_MOESM1_ESM.pdf]
